# The Use of Single-Cell Mitochondrial DNA SNP Combinations for Distinguishing Organ-Specific Cell Types

**DOI:** 10.3390/cells15100947

**Published:** 2026-05-21

**Authors:** Shuai Wang, Xinyue Tu, Haozhe Zhu, Ce Gao, Jianan Gao, Jinsong Wei, Hui Shi, Jinrong Peng

**Affiliations:** MOE Key Laboratory for Molecular Animal Nutrition, College of Animal Sciences, Zhejiang University, Hangzhou 310058, China; 12017014@zju.edu.cn (S.W.); 3210102608@zju.edu.cn (X.T.); 12217012@zju.edu.cn (H.Z.); cgao7@bwh.harvard.edu (C.G.); 3190101339@zju.edu.cn (J.G.); 3150104845@zju.edu.cn (J.W.)

**Keywords:** cell lineage, MAESTER, mitochondrial DNA, single-cell sequencing, single nucleotide polymorphism (SNP)

## Abstract

**Highlights:**

**What are the main findings?**

- Collective mitochondrial DNA SNP combinations in single cells are suitable as hallmarks for defining cell types.
- Collective mitochondrial DNA SNP combinations in single cells are suitable for cell lineage tracing during organ regeneration.

**What are the implications of the main findings?**

- Collective single-cell mitochondrial DNA SNP combinations may be used as non-invasive genetic markers for tracing cell lineages/fates during organ/tissue development or regeneration.

**Abstract:**

Cell lineage relationship studies in developmental and regenerative biology have been greatly advanced using techniques such as fluorescent labeling driven by cell-type-specific promoters. Nevertheless, unbiased non-invasive tools for distinguishing cell lineages are inevitably desired. Mitochondrial DNA (mtDNA) exhibits wide-range single-nucleotide polymorphisms (SNPs) among individual cells. Here, we aim to distinguish cell types in organs/tissues of the same individual and in the regenerated liver based on the use of mtDNA SNPs. For this, two approaches—“Mitochondrial Alteration Enrichment and Sequencing” (MAESTER) and “mitochondrial single-cell assay for transposase-accessible chromatin with sequencing” (mtscATAC-seq)—were adopted to facilitate the detection of mtDNA SNPs in single cells. With MAESTER, we show that specific cell types in the liver and spleen of the same individual can be successfully defined using collective individual-specific markers composed of panels of unique mtDNA SNP combinations. For its application, we performed partial hepatectomy (PH) on a *Krt19:Dre^ERT2/+^;R26:Rox-ZsGreen-Stop-Rox-tdTomato/+* mouse harboring tdTomato-labeled cholangiocytes following tamoxifen injection and demonstrated that utilizing panels of unique mtDNA SNP combinations detected by mtscATAC-seq in the pre-PH cholangiocytes as markers can faithfully trace the cell fate in the post-PH liver samples. Hence, this approach may serve as an unbiased tool for investigating cell lineage relationships in relevant research areas such as liver regeneration.

## 1. Introduction

Cell lineages are established during embryogenesis, organogenesis, tissue/organ regeneration/renewal, and tumorigenesis. Diverse lineage-tracing technologies have been developed. Cell-specific fluorescent labeling achieved by the tamoxifen-inducible CreER-loxP or DreER-Rox system is one of the most widely applied methods [1,2]. Multicolor reporter systems, such as Brainbow and Confetti, further expand the capabilities of lineage tracing [3,4,5]. Retroviral and adeno-associated virus (AAV) vectors have also been widely used for long-term tracking of cells [6,7]. However, these lineage-tracing technologies face notable challenges, such as limited availability of gene-specific promoters and inherent variability in promoter specificity and labeling efficiency, which have led to inconsistent results when studying the origin of cells during liver regeneration, sometimes even yielding contradictory conclusions [8,9,10,11]. Therefore, there is a strong need to develop a non-invasive method that can simultaneously track the lineage of all cells in developmental and regenerative biology.

Cell lineage tracing based on somatic genetic markers—such as single-nucleotide variants (SNVs), copy number variations (CNVs), and short tandem repeats (STRs)—is an ideal unbiased approach. However, whole-genome sequencing (WGS) at single-cell resolution, though theoretically powerful, is both costly and technically demanding, with high error rates that impose stringent requirements on variation detection algorithms [12,13,14,15]. CRISPR-Cas9-based genetic labeling is a promising solution; however, it has drawbacks such as off-target effects, elicitation of the DNA damage response, and cell apoptosis [16,17]. Although the newly reported single-cell DNA methylation sequencing method provides a powerful, endogenous, high-resolution approach to trace cell lineage, its reliability for long-term lineage reconstruction remains uncertain, as DNA methylation patterns are not strictly irreversible and can be influenced by environmental factors [18].

The use of mtDNA SNPs identified based on scRNA-seq has recently emerged as a promising, unbiased approach for cell lineage tracing [19]. Unlike nuclear DNA, mtDNA lacks the protective shields of histone modifications and damage repair tools, resulting in a mutation frequency 10 to 100 times higher than that of the nuclear genome, which can be tissue-specific. Additionally, the high copy number of mtDNA—ranging from 100 to 1000 copies per cell—ensures that even the human and mouse 16 kb mtDNA can harbor sufficient heritable variation information for lineage tracing, irrespective of cell type. Studies have shown that somatic mtDNA SNPs in humans, even at heteroplasmy levels as low as 5%, can be stably inherited across generations [20,21,22]. Approaches such as scRNA-seq, MAESTER, mtscATAC-seq, scMito-seq, LIS, and scMitoMut have been successfully applied to detect reliable mtDNA SNPs in single cells in cancer tissues and hematopoietic cells [19,23,24,25,26]. The seminal report in this field by Ludwig et al. clearly demonstrated the feasibility of using a variable number of mtDNA SNPs for the analysis of clonal or cellular relationships in various contexts, such as propagated sub-clones derived from the hematopoietic TF1 cell line, various human tissues with RNA-seq data, colonies derived from primary hematopoietic cells, sub-clonal structure in primary human colorectal cancer, and cells from chronic myelogenous leukemia (CML) patients [19]. However, these approaches have neither been applied to distinguish cell types among different solid organs of the same individual nor during organ regeneration.

In this study, based on the detection of single-cell mtDNA SNPs by MAESTER in conjunction with scRNA-seq and mtscRNA-seq, we demonstrate that the use of individual-specific collective panels of unique mtDNA three-SNP combinations can define specific cell types within the same mouse liver or spleen and trace cell lineages during liver regeneration, thus providing a proof of concept for tracing cell lineages based on mtDNA SNPs during organogenesis or organ/tissue regeneration.

## 2. Materials and Methods

### 2.1. Mouse

All animal procedures were performed in full accordance with the “Guide for the Care and Use of Laboratory Animals” issued by the Animal Ethics Committee of Zhejiang University with permission (AP CODE: ZJU20220068). Two-month-old male C57BL/6 mice were anesthetized via intraperitoneal injection of sodium pentobarbital and euthanized by cervical dislocation. Liver and spleen tissues from the same mice were dissected for subsequent experiments.

### 2.2. Single-Cell RNA Sequencing (scRNA-seq)

Mouse liver and spleen tissues from the same individuals were dissociated using a multi-enzyme cocktail (at 37 °C with an optimized digestion time). Cell viability was assessed by trypan blue exclusion, and high-viability samples were processed via the 10× Genomics Chromium system (CG000204 Chromium Next GEM Single-Cell 3′Reagent Kits v3.1). Sequencing was performed on the illumina (San Diego, CA, USA) NovaSeq 6000 platform (150 bp paired-end). Raw data were aligned to the mm10 genome “https://hgdownload.soe.ucsc.edu/goldenPath/mm10 (accessed on 15 September 2024)”, followed by the generation of the gene-cell count matrix “https://www.10xgenomics.com/support/ (accessed on 15 September 2024)”. Downstream analysis with R 4.1.3 packages (Seurat 4.2.0, dplyr 1.0.10, and patchwork 1.1.2) included quality control (200 < nFeature_RNA < 8000, percent.mt < 20%), normalization, variable feature selection, integration across samples, dimensionality reduction (PCA; npcs = 30), UMAP, and marker gene detection (FindAllMarkers, resolution = 0.8) “https://satijalab.org/seurat/ (accessed on 30 September 2024)”. Cell-type annotation was based on CellMarker, PanglaoDB, MCA, and known markers “http://117.50.127.228/CellMarker/index.html (accessed on 30 September 2024)” [27]. We employed the scCODA method to quantify cell-type proportions in the scRNA-seq data [28].

### 2.3. Mitochondrial Enrichment Sequencing (MAESTER)

Following the MAESTER method [24], we designed 105 specific primers covering the transcripts of 15 mitochondrial genes (Table S1). These 105 primers were divided into 17 groups (Table S1) for PCR amplification of the corresponding mitochondrial genes using the cDNA generated for scRNA-seq to preserve barcode and UMI information. The PCR products were purified using DNA selection beads and used as templates for the second round of PCR. During the second round of PCR, primers containing Illumina sequencing adapters were added for amplification. The PCR products were subjected to paired-end 150 bp sequencing on the NovaSeq 6000. For mitochondrial SNP detection at single-cell resolution, we employed the maegatk pipeline “https://github.com/caleblareau/maegatk (accessed on 2 April 2025)”. Raw sequencing files were initially filtered based on barcode information from the scRNA-seq analysis, followed by the removal of mitochondrial primer-binding regions. Reads were aligned to chrM in the mm10 genome and merged with scRNA-seq BAM files. SNPs were called using maegatk’s default parameters. An allele frequency matrix was constructed based on the MAESTER code, incorporating coverage and quality metrics for each SNP “https://github.com/petervangalen/MAESTER-2021 (accessed on 6 April 2025)”. Only SNPs with a quality score >30 were retained for downstream analysis. To mitigate potential amplification or sequencing biases in cell population definition, we used three SNPs to anchor each cell, with each SNP treated as a binary (presence/absence) call. According to the MAESTER method, a variant base-calling frequency of more than 1% in a single cell was adopted as the threshold for determining SNPs with confidence. The primary and secondary SNPs represented high-frequency mutations in specific liver or spleen cell populations, while the third SNP exhibited higher specificity. Together, these SNPs enabled complete discrimination of the target cell population from other cells within the same individual.

Analyses were performed using the MAESTER code and online tools “https://www.bioinformatics.com.cn/ (accessed on 30 May 2025); https://www.chiplot.online/ (accessed on 30 May 2025)” under default settings. For single-cell SNP heatmaps, clustering was applied using descending order.

### 2.4. Mitochondrial Single-Cell Assay for Transposase-Accessible Chromatin with Sequencing (mtscATAC-seq)

To enrich the proportion of cholangiocytes in liver samples, the liver tissue was subjected to in vitro perfusion and collagenase IV (Col IV) digestion to remove the majority of blood cells and immune cells, along with the extensive dissociation of hepatocytes. The remaining liver scaffold was then minced and sequentially digested with Col IV, Accutase, and trypsin, ultimately yielding a substantial population of cholangiocytes.

The mtscATAC-seq protocol was performed following the method described by Lareau et al [23], with optimizations to the standard 10× Chromium Next GEM Single-Cell ATAC Reagent Kits v1.1, including 1% formaldehyde fixation and the use of lysis buffer containing 0.1% NP40, which significantly enhanced mitochondrial DNA capture in scATAC-seq. Subsequently, the bam files generated by CellRanger-ATAC were analyzed using mgatk “http://github.com/caleblareau/mgatk (accessed on 13 October 2025)” to obtain single-cell mitochondrial DNA SNP matrices, followed by default parameter filtering “https://github.com/caleblareau/mtscATACpaper_reproducibility (accessed on 13 October 2025)”. Downstream analyses were primarily conducted using the Signac and Seurat R packages, with parameters set as dims = 2:20, reduction = “lsi”, and resolution = 0.5. For the identification of unique SNP combinations across cell types, the R code provided by DeepSeek “https://open.zju.edu.cn/Hiagent (accessed on 2 December 2025)” was employed. The workflow diagram was generated using Figdraw, ID: IYATI9144f “www.figdraw.com (accessed on 12 December 2025)”.

### 2.5. Statistical Analysis

Statistical analysis was performed using an unpaired two-tailed Student’s *t*-test. A *p*-value below 0.05 was considered to be statistically significant.

## 3. Results

### 3.1. scRNA-seq Analysis of Cell Compositions in Mouse Liver and Spleen

**Figure 1:**
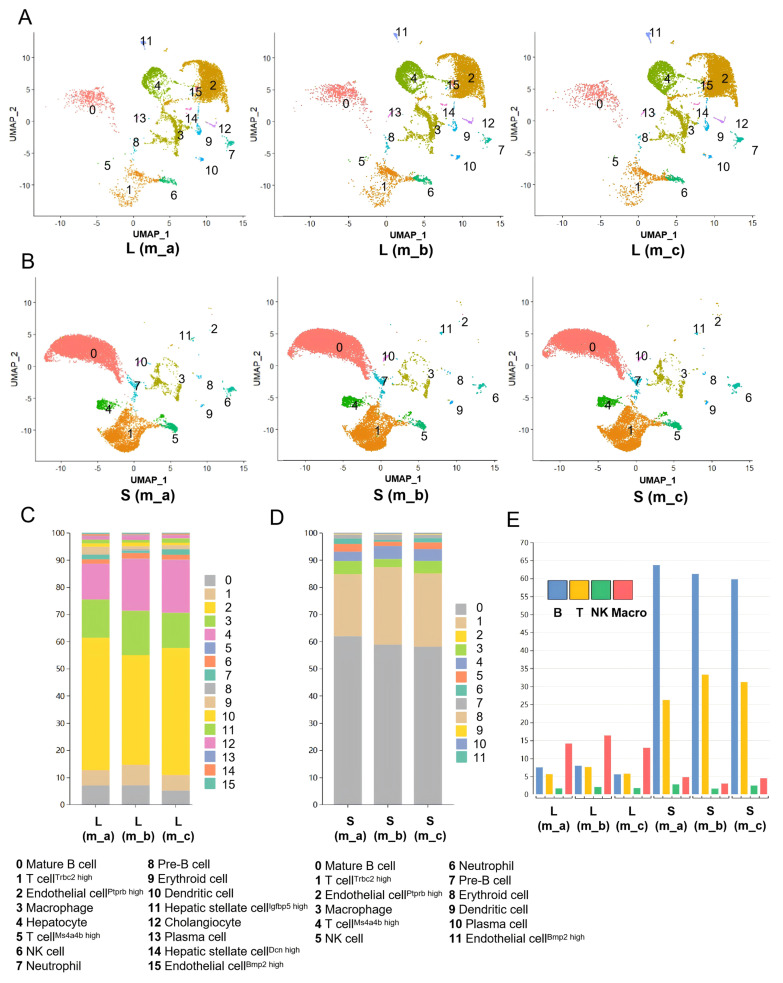
scRNA-seq analysis of cell compositions in the mouse liver and spleen. (**A**,**B**) UMAP representation of single cells sampled independently from the m_a, m_b and m_c livers (**A**) and spleens (**B**) with the annotation of cell clusters. (**C**,**D**) Comparison of ratios of each cluster of cells identified in the livers (**C**) and spleens (**D**) of m_a, m_b and m_c by scRNA-seq. (**E**) Bar chart comparing the ratios of different immune cells between the liver and spleen in m_a, m_b and m_c. B cells are in blue, T cells in yellow, NK cells in green, and macrophages (Macro) in red.

The livers and spleens from three male mice, designated as m_a, m_b, and m_c, were independently subjected to scRNA-seq on the 10xGenomics platform (data accession ID: PRJNA1363902, SUB15732715). In total, 7874, 9722 and 11,221 cells for the three livers and 14,224, 15,682 and 15,872 cells for the three spleens passed the quality control criteria based on nFeature_RNA, nCount_RNA, and Percent.mt (Supplementary Figure S1A–F). Uniform manifold approximation and projection (UMAP) analysis identified 16 and 12 cell clusters in the livers (Figure 1A) and spleens (Figure 1B), respectively. After filtering cross-contaminations, the cell clusters were annotated based on their feature genes (Supplementary Figure S2A–F). The ratios of each cluster were relatively consistent among the three livers and among the three spleens (Figure 1C,D). These findings confirmed previous reports [29,30].

The cell clusters in the liver included non-circulating cells such as hepatocytes (Cluster 4) and cholangiocytes (Cluster 12) (Figure 1A). Only one type of non-circulating cell (i.e., endothelial cells, Clusters 2 and 11) was identified in the spleen (Figure 1B). Notably, the proportions of immune cells such as macrophages, B cells, and T cells exhibited obvious differences between the liver and spleen (Figure 1C,D). Consistent with the spleen’s role in the storage of B and T cells, the spleens were composed of ~60% B cells and ~25% T cells, in contrast to ~5.5% B cells and ~5% T cells in the liver (Figure 1E).

### 3.2. mtDNA SNPs Detected by scRNA-seq Are Insufficient for Cell Lineage Tracing

**Figure 2:**
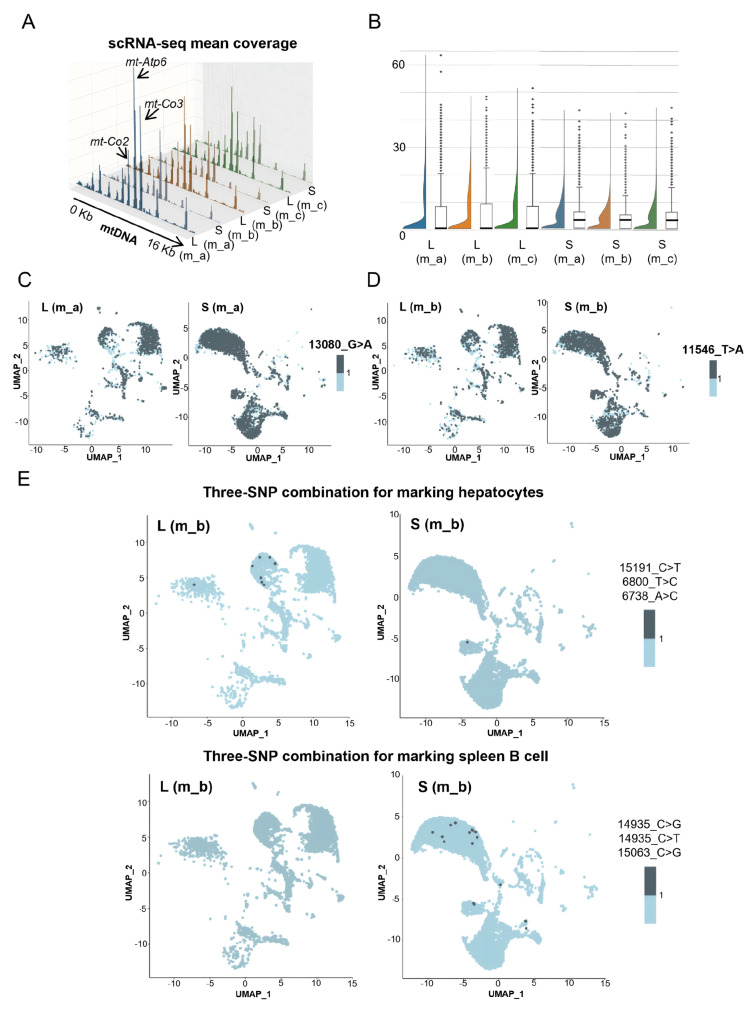
mtDNA SNPs detected by scRNA-seq are insufficient for cell lineage tracing. (**A**) 3D histogram showing the coverage of the mtDNA genome per cell through analyzing the scRNA-seq reads in the three livers and three spleens. The reads of *mt-Atp6*, *mt-Co3* and *mt-Co2* genes ranked in the top three. (**B**) Split-violin plot and statistical analysis of per-cell SNPs detected by scRNA-seq in the three livers and three spleens from m_a, m_b and m_c. (**C**,**D**) Feature plot of two representative SNPs shared by different cell types within intra-individual livers and spleens of m_a (**C**) and m_b (**D**). (**E**) Feature plot of cells in the UMAP using a hepatocyte (**top two panels**) or a spleen B cell-unique (**bottom two panels**) three-SNP combination.

The averaged mitochondrial whole genome covered by the scRNA-seq reads per cell was approximately 49.8%, 56.6% and 57.4% in the three livers and 55.9%, 57.5% and 61.4% in the three spleens, respectively. Per-cell mean reads derived from *mt-Co2*, *mt-Atp6* and *mt-Co3* genes were the most abundant in both livers and spleens (Figure 2A). Compared to the reference mtDNA genome, the per cell mean numbers of SNPs were 4.7, 4.9 and 4.5 in the three livers and 3.9, 3.6 and 3.7 in the three spleens (Figure 2B). Some SNPs (e.g., 13080_G>A in m_a and 11546_T>A in m_b) were shared across different cell types between intra-individual liver and spleen, suggesting that these SNPs were likely inherited maternally or mutated before the differentiation of the endoderm and mesoderm during embryogenesis (Figure 2C,D).

We failed to identify a unique SNP that could be used to define a specific cell type between the intra-individual liver and spleen. This is likely due to SNP inheritance from the progenitors or to the small genome size of mtDNA coupled with high mutation rates. To overcome these drawbacks, we decided to identify unique combinations composed of three distinct SNPs detected in a single cell as the hallmark for a specific cell type. Prior to this, the cells were filtered with an arbitrary threshhold of at least five SNPs per single cell as the cutoff value, and many cells were excluded from the analysis due to a lack of sufficient SNPs (Figure 2B). Through the use of an unbiased enumeration method for a thorough search, a total of 21 panels of cell-specific unique three-SNP combinations were identified in the three mice, including five panels for hepatocytes, seven panels for spleen B cells, and five panels for spleen T cells (Figure S3). However, because each panel only identified a small proportion of cells in its corresponding cell cluster (Figure 2E), this left many cells unmarked. Therefore, due to the low coverage of the mtDNA genome by scRNA-seq reads, the mtDNA SNPs detected by scRNA-seq are insufficient for thoroughly defining cell lineages.

### 3.3. MAESTER Greatly Increases the Coverage of the mtDNA Genome and the Detected Number of SNPs per Cell

**Figure 3:**
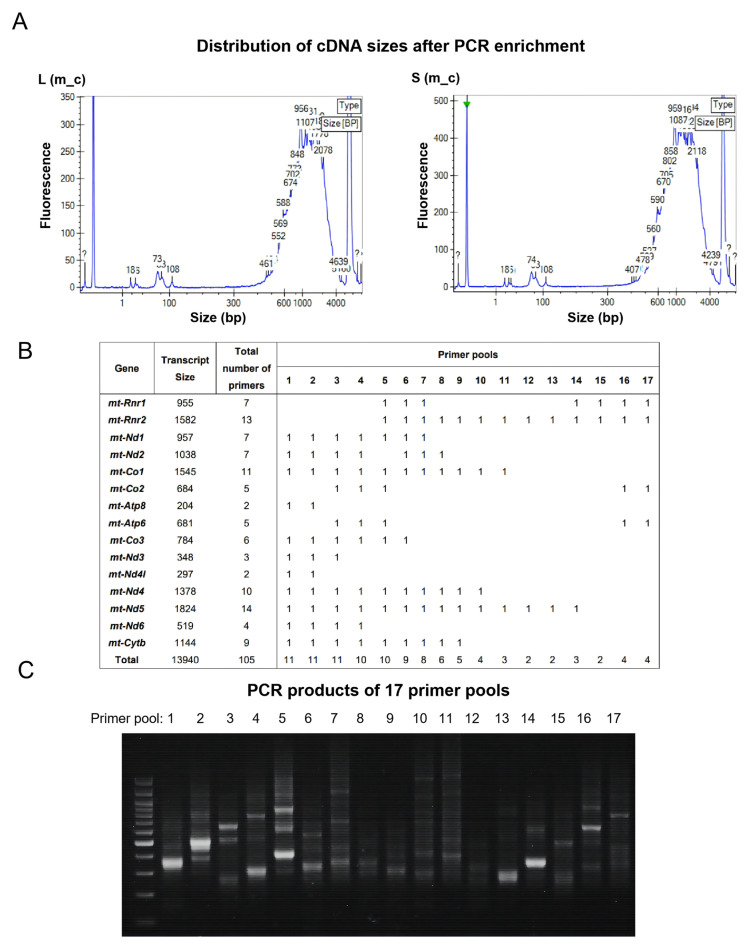
Amplification of mitochondrial gene transcripts for detecting SNPs through MAESTER. (**A**) Example showing the size ranges of mitochondrial cDNA amplified from the mouse m_c liver (**left**) and spleen (**right**) single-cell cDNA libraries as revealed by Labchip GX (Revvity, Shanghai, China). (**B**) Details of grouping 105 mitochondrial-specific primers into 17 independent pools. (**C**) An agarose gel photo showing a test example of PCR products obtained using the 17 pools of primers as the reverse primers and a universal primer covering the barcodes as the forward primer.

Next, we employed the MAESTER method to increase the coverage of the mtDNA genome. Considering that the lengths of mtDNA gene transcripts range from 204 bp to 1824 bp, to ensure the best coverage of mtDNA gene transcripts in every single cell, we enriched cDNA sizes up to 2000 bp in the cDNA libraries prepared during scRNA-seq (Figure 3A). The enriched cDNA (containing scRNA-seq barcodes) then served as the template for PCR using mtDNA gene-specific primers (105 in total) as the reverse primers paired with a universal primer (harboring barcodes) as the forward primer. The 105 primers were designed to comprehensively cover transcripts of all mtDNA genes, including two ribosomal genes (Table S1). These primers were grouped into 17 independent pools (Figure 3B), and each pool was paired with the universal forward primer for PCR. A test example showed that the sizes of the PCR products corresponded to the expected sizes for each primer pool (Figure 3C). Finally, Illumina standard adapters were added for paired-end sequencing of the PCR products [24].

**Figure 4:**
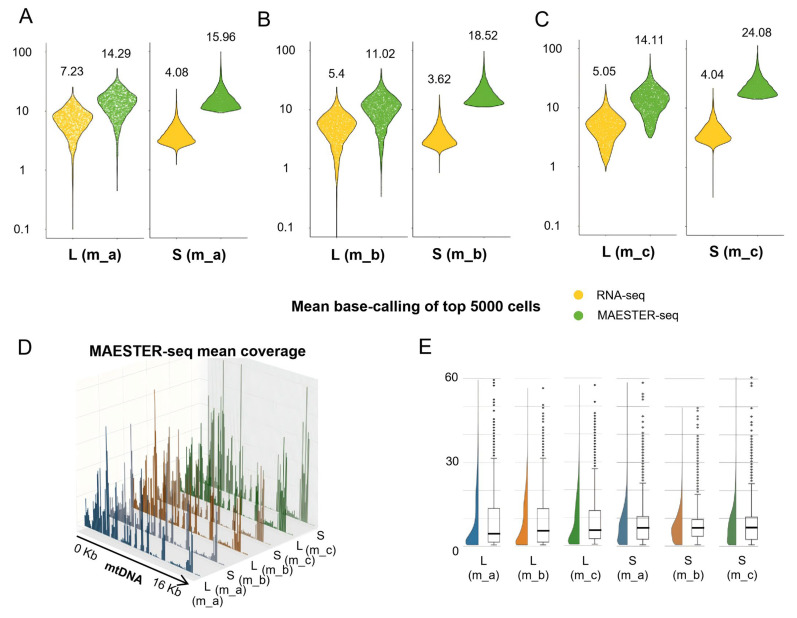
MAESTER increases coverage of the mtDNA genome per cell. (**A**–**C**) Violin graphs comparing the mean base-calling number of the mtDNA genome per cell achieved by scRNA-seq (yellow) and MAESTER-seq (green) in the top 5000 cells in the livers and spleens of m_a (**A**), m_b (**B**) and m_c (**C**). (**D**) 3D histogram showing the average coverage and base read counts across the mtDNA genome per cell for livers (L) and spleens (S) from the mice m_a (blue), m_b (brown), and m_c (green) achieved by MAESTER. (**E**) Split-violin plots and box graphs showing the number of SNPs per cell detected in the livers (L) and spleens (S) from the mice m_a (blue), m_b (brown), and m_c (green) by MAESTER.

Analysis of the filtered sequences obtained by MAESTER showed an obvious increase in the coverage of the mtDNA genome per cell in m_a, m_b and m_c, respectively (Figure 4A–C). Importantly, MAESTER significantly increased and also normalized the read counts for all mtDNA genes (Figure 4D). The Mitochondrial Alteration Enrichment and Genome Analysis Toolkit (MAEGATK) was then adopted to scrutinize mtDNA transcripts from every single cell based on barcode information to identify mtDNA SNPs at the single-cell level [24]. With respect to the mtDNA reference genome, the average number of detected SNPs per cell increased to 7.8, 7.9 and 7.8 in the three livers and 7.0, 6.5 and 7.2 in the three spleens (Figure 4E).

### 3.4. Lineage Relationships Among Cells Within the Same Cell Cluster Are Inferred by Hierarchical Clustering of the mtDNA SNPs Detected by MAESTER

**Figure 5:**
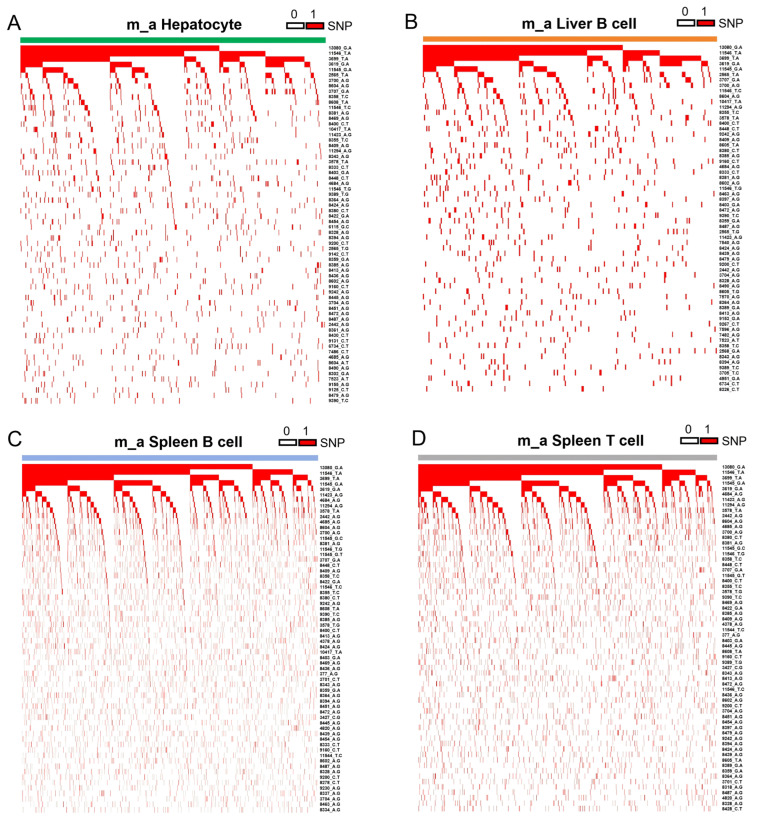
Establishing the sub-lineage relationships in the same cell cluster using mtDNA SNPs detected by MAESTER. (**A**–**D**) Hierarchical clustering heatmap based on single-cell mtDNA SNPs showing the sub-lineage relationships for hepatocytes (**A**), liver B cells (**B**), spleen B cells (**C**) and spleen T cells (**D**) in m_a.

To test whether the lineage relationships among cells within the same cell cluster might be established by clustering single-cell mtDNA SNPs, we constructed hierarchical clustering heatmaps for the clusters of hepatocytes (484 cells), liver B cells (264 cells), spleen B cells (2287) and spleen T cells (1344) using single cells harboring a minimum of five SNPs (ranging from 5 to 58) (Figure 5A–D; Table S2). In each hierarchical clustering heatmap, individual cells were auto-sorted and clustered in descending order based on the number of shared SNPs from highest to lowest using Microsoft Excel. The heatmaps displayed clear hierarchical clonal relationships among cells in each of these cell clusters (Figure 5A–D).

### 3.5. Cell Types Within the Intra-Individual Liver and Spleen Are Distinguishable Using Unique SNP Combinations Detected by MAESTER as Markers

**Figure 6:**
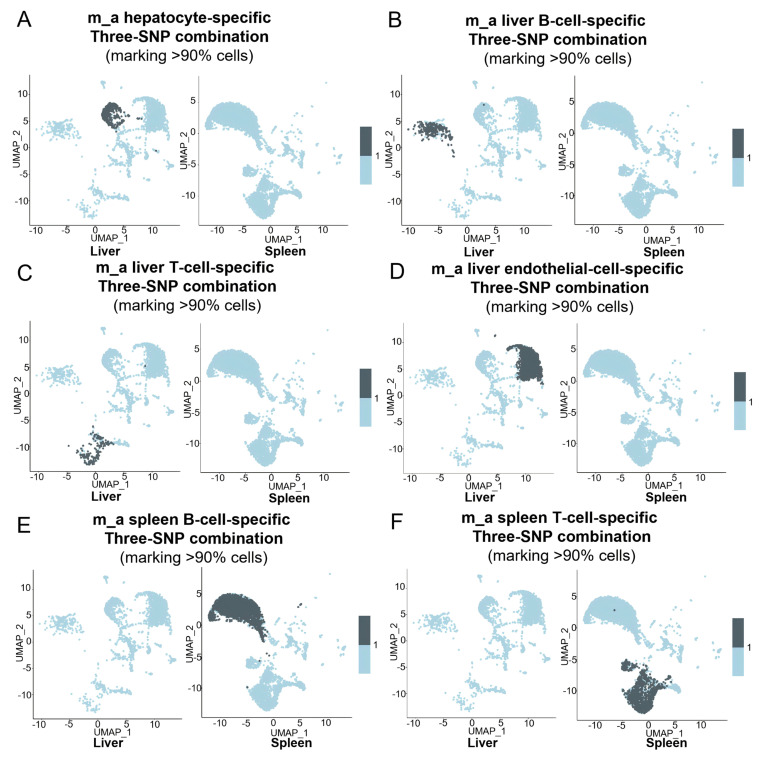
Using unique three-SNP combinations detected by MAESTER to define specific cell types in the liver or spleen. (**A**–**F**) Feature plots showing >90% cells marked by the panels of unique three-SNP combinations for hepatocytes (**A**), liver B cells (**B**), liver T cells (**C**), liver endothelial cells (**D**), spleen B cells (**E**), and spleen T cells (**F**) in the UMAPs of m_a liver and spleen.

One of our goals was to use unique mtDNA SNPs to define different cell types/clusters within an organ. Since it was almost impossible to use a single mtDNA SNP to define a specific cell type unequivocally, we again tried to use individual-specific panels of unique three-SNP combinations as the hallmarks to define intra-individual liver- and spleen-specific cell types. Based on the SNPs listed in an Excel sheet (Table S2), we identified 124 panels for 484 hepatocytes (Figure S4), 73 panels for 264 liver B cells (Figure S5), 466 panels for 2287 spleen B cells (Figure S6), 54 panels for 177 liver T cells (Figure S7) and 294 panels for 1344 spleen T cells (Figure S8) in m_a. Collectively, panels for each specific cell type marked >90% of the cells in their corresponding cell clusters. Feature plotting showed that m_a hepatocyte-unique three-SNP combinations specifically marked hepatocytes (Figure 6A). Panels specific for m_a liver B, T and endothelial cells successfully identified their corresponding cell types in the liver (Figure 6B–D). Meanwhile, panels specific for liver B, T and endothelial cells were largely negative in spleen B, T and endothelial cells (Figure 6B–D). Similarly, panels specific for m_a spleen B and T cells marked the corresponding cell types in the spleen but were largely negative in liver B and T cells (Figure 6E,F).

**Figure 7:**
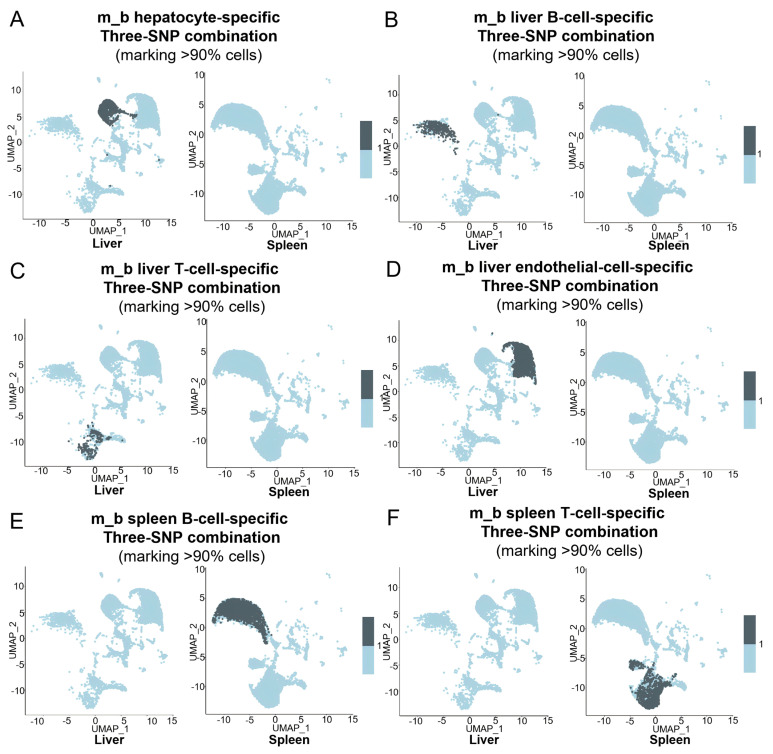
Using unique three-SNP combinations detected by MAESTER to define specific cell types in the liver or spleen. (**A**–**F**) Feature plots showing >90% cells marked by the panels of unique three-SNP combinations for hepatocytes (**A**), liver B cells (**B**), liver T cells (**C**), liver endothelial cells (**D**), spleen B cells (**E**), and spleen T cells (**F**) in the UMAPs of m_b liver and spleen.

**Figure 8:**
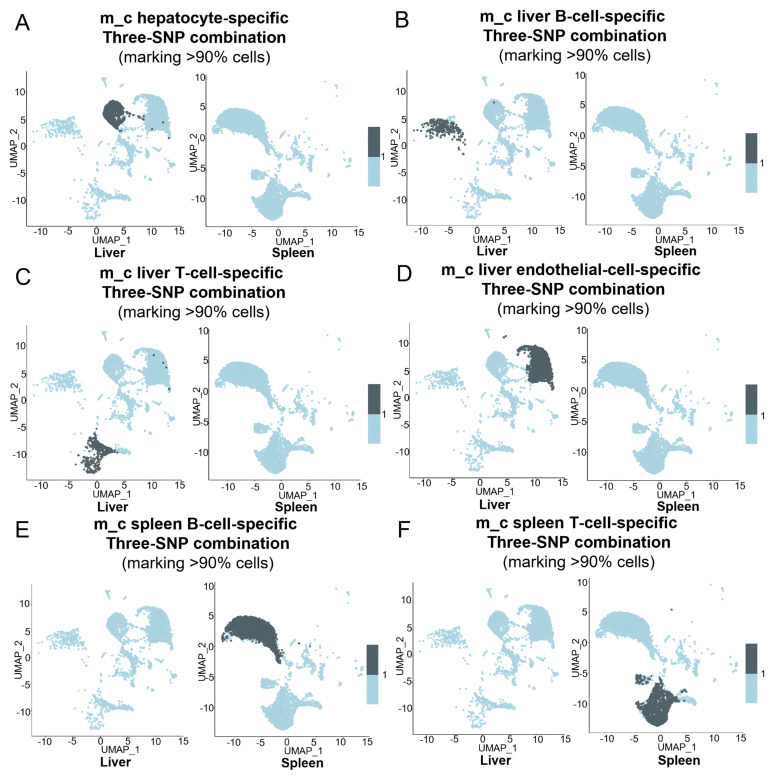
Using unique three-SNP combinations detected by MAESTER to define specific cell types in the liver or spleen. (**A**–**F**) Feature plots showing the >90% cells marked by the panels of unique three-SNP combinations for hepatocytes (**A**), liver B cells (**B**), liver T cells (**C**), liver endothelial cells (**D**), spleen B cells (**E**), and spleen T cells (**F**) in the UMAPs of m_c liver and spleen.

Similarly, panels of unique three-SNP combinations specific for liver and spleen cell types clearly marked their corresponding organ/tissue-specific cell types in the m_b (Figure 7A–F) and m_c (Figure 8A–F). Taken together, these results demonstrate that the collective use of panels of unique three-SNP combinations can define specific cell types in an organ.

### 3.6. mtscATAC-seq Achieves Almost 100% Coverage of the Mitochondrial Genome and Better Detection of mtDNA SNPs in Single Cells

**Figure 9:**
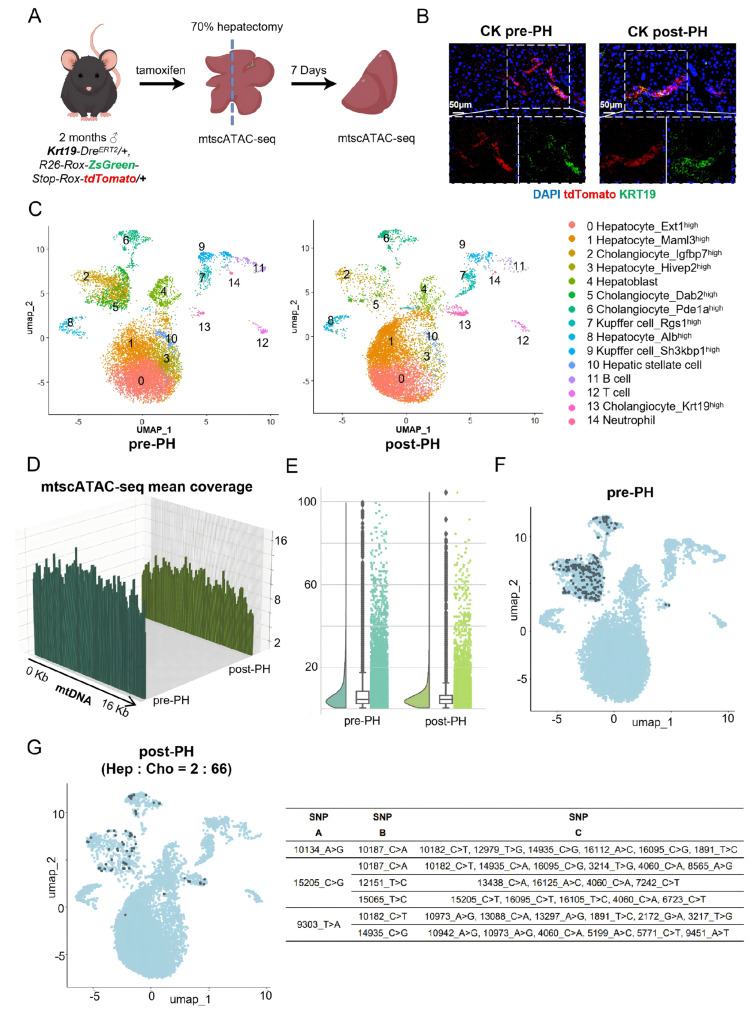
Panels of unique mtDNA SNP combinations as collective markers faithfully trace the fate of *Rox-ZsGreen-Stop-Rox* excised cholangiocytes in the regenerated liver. (**A**) A diagram outlining the scheme of the cell lineage tracing study using the “*Krt19-Dre^ERT2/+^;R26-Rox-ZsGreen-Stop-Rox-tdTomato/+*” mouse to generate tdTomato-labeled cholangiocytes upon tamoxifen injection, followed by 70% PH. (**B**) Co-immunostaining of tdTomato and KRT19 in the pre-PH and post-PH liver samples. (**C**) UMAP representation of single cells from the pre-PH and post-PH liver samples. The genomic ATAC-seq peaks extracted from the mtscATAC-seq data were used to construct the UMAP. Cell cluster annotations are on the right. (**D**) 3D histogram showing the coverage of the mtDNA genome per cell achieved by mtscATAC-seq. (**E**) Split-violin plot and box statistical analysis of per-cell SNPs detected by mtscATAC-seq in the pre-PH and post-PH liver samples. (**F**) Feature plot of panels of unique SNP combinations collectively marking >90% of the *Rox-ZsGreen-Stop-Rox* excised cholangiocytes on the UMAP of the pre-PH liver sample. (**G**) Plotting of panels of unique SNP combinations in (**F**) onto the UMAP of the post-PH liver sample. Details of the panels of unique three-SNP combinations are shown on the right.

Next, we decided to examine whether the panels of unique mtDNA three-SNP combinations could be used as markers for tracing cell lineages during liver regeneration. To facilitate cell lineage tracing, we carried out this experiment in the mouse harboring the *Krt19:Dre^ERT2/+^;R26:Rox-ZsGreen-Stop-Rox-tdTomato/+* transgene. In this mouse line, tamoxifen injection results in the excision of *Rox-ZsGreen-Stop-Rox* by the Dre recombinase, leading to cholangiocytes being labeled by tdTomato (Figure 9A), thus allowing us to trace the fate of existing cholangiocytes after liver regeneration.

One *Krt19:Dre^ERT2/+^;R26:Rox-ZsGreen-Stop-Rox-tdTomato/+* mouse (2 months old) was subjected to 70% PH and allowed to regenerate its liver for one week. Then, the pre-PH (the resected liver lobe) and post-PH (liver tissue collected one week after the operation) samples were assayed for the expression of tdTomato together with immunostaining of the bile duct marker keratin 19 (KRT19). The results showed that tdTomato signals co-localized with KRT19 signals in both pre-PH and post-PH samples (Figure 9B), demonstrating that, in a normal mouse, the cholangiocytes in the regenerated liver were mainly derived from pre-existing cholangiocytes.

Because MAESTER only allows for the detection of mtDNA SNPs in the 3′ end of the gene coding region and requires a complicated experimental procedure, we adopted mtscATAC-seq, which is presumably able to cover 100% of the mitochondrial genome [23], to detect mtDNA SNPs. The above pre-PH and post-PH liver samples were subjected to mtscATAC-seq. After quality assessment (Figure S9), genomic ATAC-seq peaks were used to construct a UMAP for cell clustering, and a total of 15 cell clusters were defined (Figure 9C). Cell clusters were then annotated based on the ATAC-seq peaks corresponding to cell-specific feature genes (Figure 9C; Supplementary Figure S10A–F).

### 3.7. The Use of Panels of Unique mtDNA SNP Combinations as Markers Faithfully Traces the Fate of Rox-ZsGreen-Stop-Rox-Excised Cholangiocytes in the Regenerated Liver

To explore whether panels of unique mtDNA SNP combinations could be used as markers to trace cell lineages during liver regeneration, we used the mtscATAC-seq data to extract the mtDNA information. We found that mtscATAC-seq achieved almost 100% coverage of the mitochondrial genome in individual cells (Figure 9D) and, importantly, greatly increased the number of SNPs (up to 101 SNPs) detected at single-cell resolution (Figure 9E).

We then identified the subpopulation of cholangiocytes in the pre-PH liver by checking the feature sequence left by the excision of *Rox-ZsGreen-Stop-Rox* (i.e., expected to be tdTomato-positive) (Figure S11A), and a total of 295 such cells were identified (Figure 9F). Next, using the software generated by DeepSeek (https://open.zju.edu.cn/Hiagent), panels of unique mtDNA three-SNP combinations collectively marking >90% of the *Rox-ZsGreen-Stop-Rox*-excised cholangiocytes in the pre-PH samples were established (Figure 9G). Then, we searched for cells that harbored the above pre-PH unique three-SNP combinations markers in the single-cell UMAP of the post-PH liver, and a total of 142 cells were identified (Figure 9G). Importantly, 68 of them were found to carry the *Rox-ZsGreen-Stop-Rox* excised sequence. When checking the distribution of these cells, we found that the majority were within the clusters 2, 5, 6 and 13, annotated as cholangiocytes, while two cells were found in cluster 1, which was annotated as hepatocytes (Figure 9G). In addition, we also searched for the panels of unique three-SNP combinations collectively covering over 90% of hepatocytes in the pre-PH liver (Figure S11B,C) and then checked the existence of these panels in the single-cell UMAP of the post-PH liver. A total of 188 cells were identified, and 62, 55, 9, and 62 of these cells were within clusters 0, 1, 3 and 8, respectively, which were annotated as hepatocytes (Figure S11D), confirming that, in a normal mouse, the regenerated hepatocytes are derived from pre-existing hepatocytes [10]. Taken together, these results demonstrate that panels of unique mtDNA SNP combinations can be used as collective markers for cell lineage tracing during liver regeneration.

## 4. Discussion

During early organogenesis, germ cells systematically differentiate into different cell types to exert specific functions in an organism. During cell renewal or replenishment in adult organs/tissues such as the skin and intestinal epithelium, stem cells differentiate into functional cell types. During organ regeneration after damage (e.g., by a toxin or surgical operation), relevant cells reenter the cell cycle to compensate for the loss of organ/tissue mass. All of these processes involve cell lineage specification. The development of an unbiased approach to trace cell lineage relationships in these processes is a longstanding challenge, although tremendous progress has been achieved [31,32,33,34,35,36,37]. Our main goal was to use mtDNA SNPs for cell lineage tracing during organ (such as liver) regeneration, an approach that has not been tested previously. For this, we first adopted the MAESTER method to detect mtDNA SNPs. Since existing research on using mtDNA SNPs for cell lineage tracing primarily focused on cultured cells, cancer tissues, hematopoietic stem cells and immune cells (such as in the MAESTER report), we needed to test its suitability in distinguishing cell types in solid organs/tissues prior to applying this method to study organ regeneration. During the course of our study, we found that it is almost impossible to use a single mtDNA SNP to define a specific cell type, which is attributed to the high mutation rate of mtDNA during the development of an organism. In addition, the use of a single mtDNA SNP might encounter errors caused by reverse transcription, PCR or sequencing that would complicate cell-type definition. We found that the use of cell-type-specific panels of unique mtDNA three-SNP combinations as collective markers allows us to define specific cell types. Considering that errors occurring simultaneously in three nucleotides in a group of cells are extremely rare, we are fairly confident of the suitability of this method for studying cell lineage relationships in vivo.

Our pilot experiment, described in Figure 9, suggests that the individual-derived specific panels of unique three-SNP combinations of mtDNA are particularly suitable for studying liver regeneration after partial hepatectomy (PH) under different stress treatments. Numerous studies have shown that while a healthy normal liver after PH regains its mass mainly through the proliferation of pre-existing hepatocytes, non-hepatocytes (such as cholangiocytes) could contribute to liver regeneration when the hepatocytes harbor a genetic defect in proliferation or survival [10,38,39,40,41]. In the latter case, fluorescent labeling assisted by specific promoters has made impressive progress in tracing the cell origin of liver regeneration; however, such approaches normally can only trace the fate of one unique type of cell [42,43]. Thus far, there is a lack of an approach that could thoroughly examine hepatocyte identities after regeneration. For mtDNA SNP-assisted cell lineage tracing, the scenario is that one can use the resected portion of the liver to perform scRNA-seq and MAESTER simultaneously or mtscRNA-seq. Next, panels of unique three-SNP combinations for all cell types are identified based on mtDNA SNPs. Then, the regenerated liver of the same individual is sampled and subjected to scRNA-seq and MAESTER or mtscRNA-seq. Finally, by checking the distributions of the previously identified panels of unique three-SNP combinations within the single cells in the regenerated liver, one can infer the cell origin of the regenerated hepatocytes or cholangiocytes.

Despite the potential use of mtDNA SNPs in defining cell types, we find that some drawbacks need to be kept in mind. First, the high mutation rate of mtDNA disallows the use of a single mtDNA SNP in defining a specific cell type. Second, MAESTER greatly increases the coverage of the mtDNA genome; however, performing MAESTER involves multiple steps and is technically demanding. Third, the MAESTER approach is based on detecting the 3′ end of gene transcripts, so it can only provide information from a limited mtDNA coding region. Fourth, although the mtscATAC-seq approach broadens the coverage of the mitochondrial genome and increases the chances of detecting mtDNA SNPs, it has the disadvantage of difficulty in cell clustering. Fifth, due to mitophagy, some ancestral mtDNA SNPs might be lost in a cell, thereby causing difficulty in tracing cell lineage. In conclusion, our data provided proof of concept for adopting single-cell mtDNA SNP combinations as an unbiased approach for defining cell types and tracing cell lineages during organ regeneration.

## 5. Conclusions

This study adopted the “Mitochondrial Alteration Enrichment and Sequencing” (MAESTER) for detecting mtDNA SNPs in single cells in the liver and spleen of three individual mice and found that specific cell types in the liver and spleen of the same individual can be successfully defined using collective, individual-specific markers composed of panels of unique mtDNA SNP combinations. This study also adopted the “mitochondrial single-cell assay for transposase-accessible chromatin with sequencing” (mtscATAC-seq) approach for detecting mtDNA SNPs in a mouse liver before (pre-) and after (post-) partial hepatectomy (PH). Taking advantage of mice carrying the transgene *Krt19:Dre^ERT2/+^;R26:Rox-ZsGreen-Stop-Rox-tdTomato/+*, which can label cholangiocytes with tdTomato upon tamoxifen injection, this study successfully demonstrated that utilizing panels of unique mtDNA SNP combinations detected by mtscATAC-seq in pre-PH cholangiocytes as markers can faithfully trace cell fate in the post-PH liver sample. Hence, this study establishes an unbiased tool for investigating cell lineage relationships in relevant research areas such as liver regeneration.

## Data Availability

The data presented in this study are openly available in the Sequence Read Archive (SRA) in the National Center for Biotechnology Information (NCBI) with references number [PRJNA1363902, SUB15732715].

## Supplementary Materials

The following supporting information can be downloaded at https://www.mdpi.com/article/10.3390/cells15100947/s1, Figure S1: Quality analysis of the scRNA-seq data; Figure S2: Feature genes for annotating cell clusters in the liver and spleen; Figure S3: A table summarizing all panels of unique three-SNP combinations for hepatocytes, liver endothelial cells, and spleen B and T cells identified through analyzing scRNA-seq reads in m_a, m_b and m_c; Figure S4: List of panels of unique three-SNP combinations marking >90% of hepatocytes in m_a; Figure S5: List of panels of unique three-SNP combinations marking >90% of liver B cells in m_a; Figure S6: List of panels of unique three-SNP combinations marking >90% of spleen B cells in m_a; Figure S7: List of panels of unique three-SNP combinations marking >90% of liver T cells in m_a; Figure S8: List of panels of unique three-SNP combinations marking >90% of spleen T cells in m_a. Figure S9: Quality assessment of the mtscATAC data. Figure S10: Plotting of feature genes on the mtscATAC UMAP. Figure S11: Tracing the fates of pre-PH hepatocytes in the post-PH liver. Table S1: 105 primer sequences used in MAESTER; Table S2: Summary of the number of cells with a minimum of 5 SNPs in different cell clusters.

## Abbreviations

The following abbreviations are used in this manuscript:

MAESTER	mitochondrial alteration enrichment and sequencing
mtDNA	mitochondrial DNA
mtscRNA-seq	mitochondrial single-cell assay for transposase-accessible chromatin with sequencing
scRNA-seq	Single-cell RNA sequencing
SNPs	Single-nucleotide polymorphism
