# Supplementary material for: The Use of Single-Cell Mitochondrial DNA SNP Combinations for Distinguishing Organ-Specific Cell Types
Source: Cells. 2026 May 21;15(10):947. doi: 10.3390/cells15100947 (PMC13205045; doi:10.3390/cells15100947)
Supplement: Supplementary file 1 [file cells-15-00947-s001.zip › cells-4304330-supplementary.pdf]

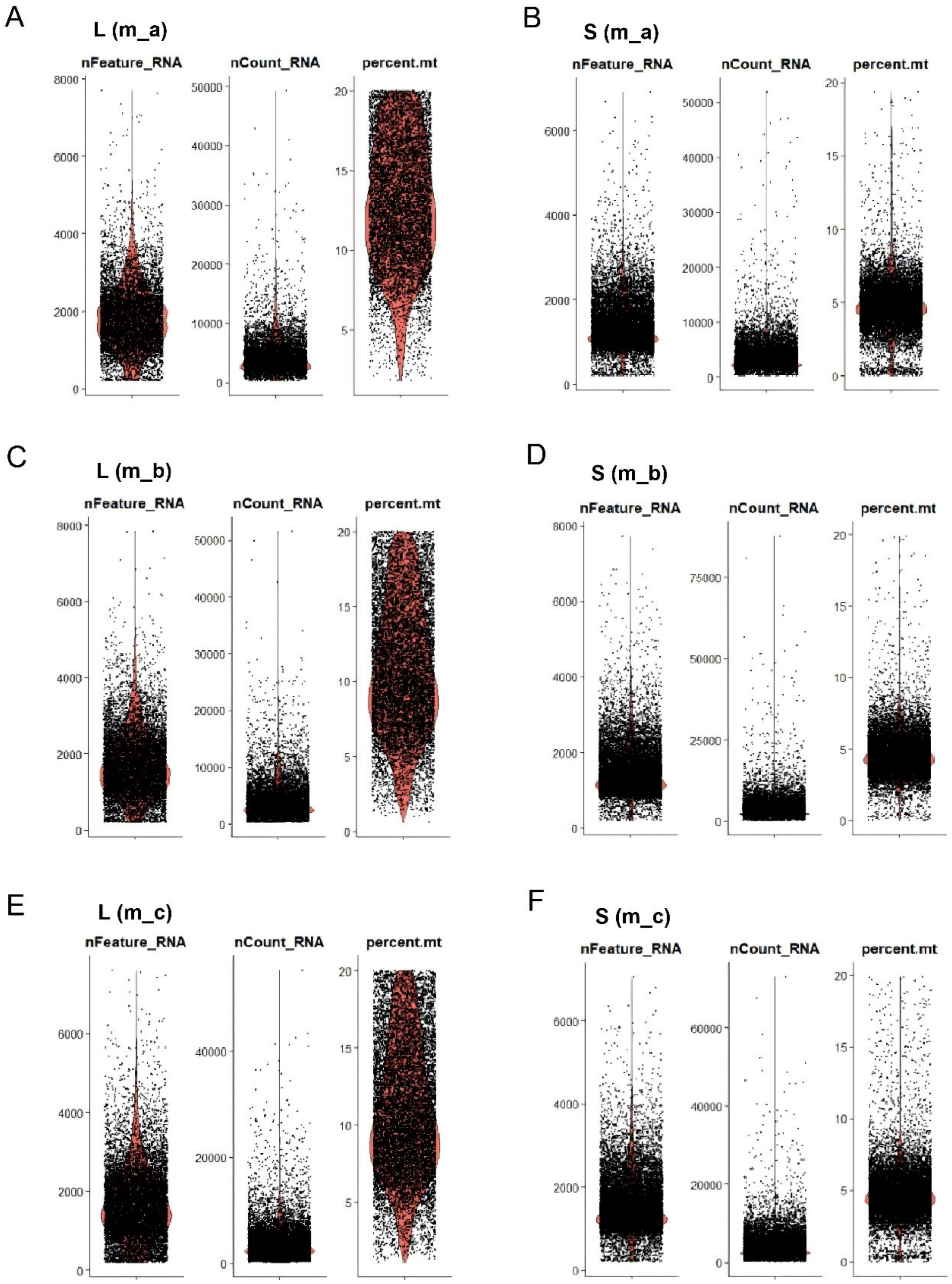

Figure S1. Quality analysis of the scRNA-seq data. (A to F) Graph showing the identified unique gene number (nFeature_RNA) (left), captured transcript number (nCount_RNA) (middle) and mitochondrial genes percentage (Percent.mt) (right) in the livers and spleens dissected from m_a (A and B), m_b (C and D) and m_c (E and F).

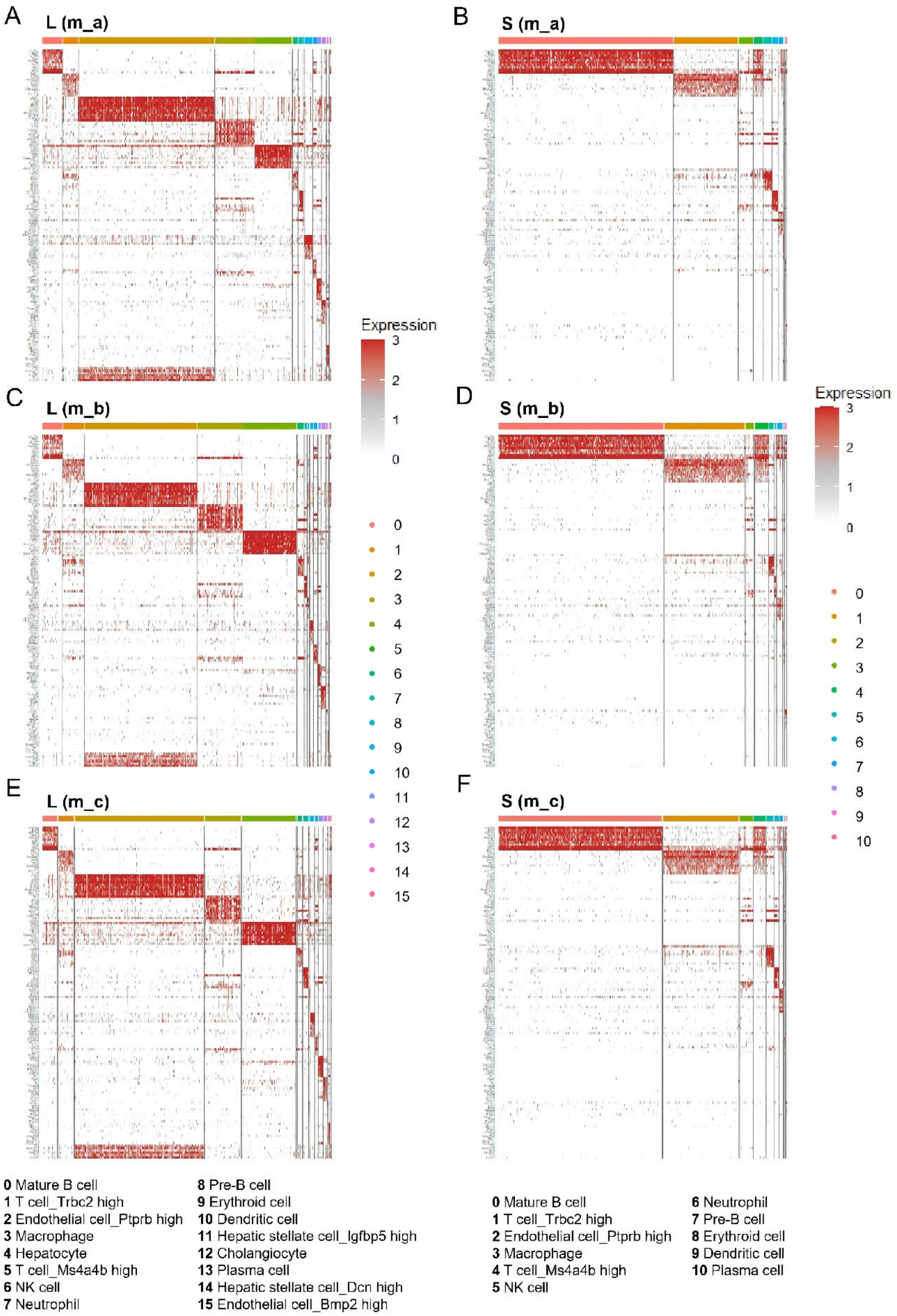

Figure S2. Feature genes for annotating cell clusters in the liver and spleen. (A to F) Heatmap showing feature genes for annotating cell clusters in the livers and spleens from m_a (A and B), m_b (C and D) and m_c (E and F). Annotation of cell clusters in the liver and spleen were provided at the bottom.

Panels of three-SNP combinations through scRNA-seq

Cell type	Sample	Panels of unique three-SNP combinations	Cell # / Total
Endothelial cell	L (m_a)	13080_G>A, 6723_C>T, 3619_G>A	9/1671
	L (m_a)	13080_G>A, 6723_C>T, 11326_C>T	8/1671
	L (m_b)	11530_C>T, 6693_A>C, 6710_A>C	12/1918
	L (m_c)	8565_A>G, 11269_C>G, 15200_A>G	15/2663
Hepatocyte	L (m_a)	6723_C>T, 3619_G>A, 11326_C>T	5/484
	L (m_b)	11530_C>T, 15191_C>T, 6800_T>C	7/843
	L (m_b)	11530_C>T, 6800_T>C, 6705_A>C	6/843
	L (m_b)	15191_C>T, 6800_T>C, 6738_A>C	7/843
	L (m_c)	8565_A>G, 15049_C>T, 11337_T>C	13/1002
B cell	S (m_a)	8565_A>G, 14935_C>G, 15050_C>A	16/2287
	S (m_a)	15051_C>T, 15125_C>T, 15050_C>A	11/2287
	S (m_b)	14935_C>T, 13737_C>G, 14935_C>G	10/1743
	S (m_b)	14935_C>G, 14935_C>T, 15063_C>G	12/1743
	S (m_b)	14935_C>T, 15063_C>G, 15134_C>A	9/1743
	S (m_c)	14935_C>T, 15058_C>G, 7368_A>G	15/2500
	S (m_c)	14935_C>T, 15058_C>G, 7415_C>A	13/2500
T cell	S (m_c)	14935_C>T, 15087_C>A, 9344_C>A	8/1741
	S (m_c)	14935_C>T, 15058_C>G. 7524_A>C	9/1741
	S (m_c)	15087_C>A, 7524_A>C, 6688_C>A	5/1741
	S (m_c)	14935_C>T, 15087_C>A, 7524_A>C	8/1741
	S (m_c)	14935_C>T, 15087_C>A, 7501_A>C	6/1741

Figure S3. A table summarizing all panels of unique three-SNP combinations for hepatocytes, liver endothelial cells, spleen B and T cells identified through analyzing scRNA-seq reads in m_a, m_b and m_c.

Panels of unique three-SNP combinations marking >90% of m_a hepatocytes

SNP A	SNP B	SNP C
2565_T>A	3699_T>A	11543_A>G, 10417_T>G, 922_T>C, 4701_A>G, 7463_C>T, 3618_T>A, 6518_C>T, 9065_A>G, 7593_G>A, 6628_C>T, 7510_C>T, 8278_C>T, 9345_G>A, 1778_T>A, 535_A>G, 9142_C>T, 8445_A>G, 8293_A>G, 8541_A>G, 6617_C>T, 11286_A>G, 7554_A>G, 7397_A>G
	11546_T>A	7397_A>G, 8290_A>G, 8501_C>T, 7554_A>G, 8265_A>G, 11286_A>G, 11291_A>G, 11299_A>T, 6617_C>T, 6691_C>T, 7512_C>T, 8377_A>G, 9977_A>C, 3704_A>G, 8328_A>G, 7450_A>T, 11334_A>G, 922_T>C, 9345_G>A, 9065_A>G, 11543_A>G
3699_T>A	3619_G>A	7620_A>G, 8475_T>A, 2416_A>T, 2516_A>T, 3599_T>A, 6844_A>G, 3438_A>T, 10030_A>G, 6563_C>T, 755_A>G, 8427_A>T, 11303_A>T, 922_T>G, 505_A>G, 587_A>T, 655_C>T, 827_A>G, 448_A>T, 964_G>A, 2372_A>T, 6836_C>T, 15053_A>T, 2460_A>T, 2683_T>A, 9804_T>C, 4679_C>T, 4855_A>T, 4726_A>T, 4801_A>T, 6714_A>T, 7394_C>T, 7455_T>A, 7516_A>T, 7524_A>T, 7561_A>T, 8287_C>T, 8349_T>A, 8414_A>T, 8416_A>T, 8441_A>T, 9095_A>T, 9283_A>T, 9620_A>T
	11546_T>A	922_T>G, 886_A>G, 10030_A>G, 8344_A>T, 10285_C>T, 3448_T>A, 7506_T>A, 7559_A>T, 7561_A>T, 9088_A>T, 1024_T>C, 10391_A>G, 13753_A>T, 2500_T>A, 2501_A>T, 2516_A>T, 2666_A>G, 3438_A>T, 3617_A>T, 399_A>G, 4726_A>T, 6659_A>T, 6737_T>A, 6795_A>T, 7410_T>A, 7530_A>T, 8330_T>A, 8405_T>A, 8531_T>A, 9002_T>A, 9060_A>T, 9081_A>T, 9137_A>T, 9194_A>T, 9202_T>A, 9265_T>A, 9301_A>T, 9384_G>T

Figure S4. List of panels of unique three-SNP combinations marking >90% of hepatocytes in m_a.

Panels of unique three-SNP combinations marking >90% of m_a liver B cells

SNP A	SNP B	SNP C
2565_T>A	3699_T>A	6944_G>A, 11318_A>T, 12562_T>A, 12826_T>A, 2060_A>G, 2494_A>T, 2566_A>T, 3572_A>T, 3598_A>T, 4691_T>A, 637_A>G, 6754_T>A, 6787_A>G, 7567_A>T, 8391_A>T, 8995_C>T, 9065_A>T, 9194_A>T
	11546_T>A	1006_A>G, 10229_A>G, 11290_T>A, 11318_A>T, 12826_T>A, 1901_T>A, 2566_A>T, 2612_A>G, 2637_A>G, 3608_A>T, 4405_A>T, 454_A>G, 567_A>G, 602_T>A, 6361_T>A, 646_G>C, 6599_A>T, 6754_T>A
3699_T>A	3619_G>A	10202_A>G, 11318_A>T, 2497_T>A, 6622_G>C, 6862_A>G, 7506_T>A, 7567_A>T, 7671_A>G, 8391_A>T, 8499_T>A, 9341_A>T, 9701_A>G
	3700_A>G	15042_A>T, 298_T>A, 3580_A>T, 489_T>G, 6708_T>A, 6754_T>A, 6795_A>T, 6844_A>G, 9060_A>T, 9062_A>T, 910_C>T, 9194_A>T, 9199_T>A, 9279_T>A, 9283_A>T, 9486_A>T, 9804_T>C
	11546_T>A	10620_T>C, 13494_A>T, 2372_A>T, 2497_T>A, 6708_T>A, 7690_A>G, 8313_T>A, 910_C>T

Figure S5. List of panels of unique three-SNP combinations marking >90% of liver B cells in m_a.

Panels of unique three-SNP combinations marking >90% of m_a spleen B cells

SNP A	SNP B	SNP C
11545_G>A	4684_A>G	11297_A>G, 2493_A>T, 8469_A>T, 10416_C>T, 2104_T>A, 2625_A>G, 3479_A>T, 4822_A>G, 500_A>G, 5015_T>G, 7483_A>G, 7569_C>A, 7631_A>G, 8269_A>T, 8446_A>G, 9065_A>G, 9263_A>G, 1025_C>A, 11012_A>T, 1240_A>T, 12634_T>A, 13895_T>A, 14146_T>A, 14928_C>G, 14950_A>G, 14956_A>G, 14998_C>T, 15175_A>T, 15246_A>G, 15285_C>T, 15341_T>G, 2175_T>A, 2501_A>G, 2612_A>G, 3536_A>T, 3639_A>G,
	11423_A>G	7605_A>G, 9092_C>T, 11424_A>T, 9295_A>G, 10416_C>T, 4819_A>G, 5936_C>T, 7389_A>G, 7582_G>T, 9145_A>T, 10295_A>G, 1240_A>T, 14660_A>T, 15341_T>A, 2079_C>T, 2218_C>T, 2535_C>G, 4676_C>T, 6551_A>G, 7320_A>G, 7547_C>T, 8950_G>A, 9068_T>A, 9143_A>T, 9168_A>T, 9183_T>A, 9294_A>T, 10220_A>G, 11133_T>A, 11390_A>T, 11544_T>A, 15105_A>T, 15153_T>A, 15251_A>G, 15965_G>A, 1781_C>T
	3578_T>A	7534_C>T, 7471_A>T, 11294_A>T, 11424_A>T, 14705_T>A, 14753_A>T, 2218_C>T, 2269_T>A, 2493_A>T, 2585_A>G, 4819_A>G, 7389_A>G, 7392_A>G, 8371_C>T, 9098_A>T, 9298_A>G, 11468_A>G, 14734_T>A, 14870_A>G, 14950_A>G, 14956_A>G, 15102_A>G, 15153_T>A, 15245_A>G, 15341_T>A, 2423_A>T, 2430_C>T, 2436_T>C, 2535_C>G, 2625_A>G, 3632_A>G, 439_A>G, 4679_C>T, 478_A>G, 567_A>G, 5936_C>T
	4378_A>G	10417_T>A, 14705_T>A, 11525_A>G, 4725_T>A, 7457_C>T, 8186_T>C, 8328_A>G, 8605_T>C, 8606_A>G, 9155_A>G, 9221_A>G, 9243_A>G, 10417_T>G, 11011_T>A, 11446_A>G, 11451_T>A, 11542_T>A, 11543_A>G, 1240_A>T, 14930_A>G, 15000_A>G, 2442_A>T, 3706_A>G, 4377_T>A, 4687_A>G, 6636_C>T, 6673_C>T, 7389_A>G, 7397_A>G, 7417_G>A, 7498_A>G, 7510_C>T, 7526_A>G, 7537_C>T, 7569_C>A, 8283_C>T
	4378_A>T	11546_T>C, 8394_A>G, 11545_G>T, 3701_C>T, 377_A>G, 6650_A>G, 6813_C>T, 7610_T>C, 8333_C>T, 8337_A>G, 8359_G>A, 8389_G>A, 8428_C>T, 9172_C>T, 9283_A>G, 9292_C>T, 9389_T>G
	6944_G>A	14962_A>T, 7486_C>T, 8409_A>G, 8551_A>G, 1006_A>G, 10406_C>T, 11424_A>T, 11434_T>A, 11443_T>A, 11546_T>G, 14753_A>T, 14950_A>G
4684_A>G	11423_A>G	8327_C>T, 14930_A>G, 15288_T>C, 3428_A>T, 3705_T>C, 4701_A>G, 6673_C>T, 7427_G>A, 7593_G>A, 7631_A>G, 8377_A>G, 8606_A>G, 9135_C>T, 9210_G>T, 9317_C>T, 1025_C>A, 10425_G>A, 11294_A>T, 11424_A>T, 14870_A>G, 15089_A>G, 15109_A>G, 1781_C>T, 392_A>G, 4822_A>G, 5015_T>G, 6563_C>T, 7388_A>G, 7392_A>G, 7530_A>G
	3619_G>A	3703_T>A, 9210_G>T, 14928_C>G, 4831_A>G, 534_A>G, 7631_A>G, 8488_A>G, 11440_A>G, 13378_T>A, 15020_A>G, 15175_A>T, 15245_A>G, 2071_C>T, 2104_T>A, 2221_A>G, 2397_A>T, 2501_A>G, 3217_T>A, 3479_A>T, 439_A>G, 4949_T>C, 517_C>T, 6361_T>A, 6638_A>G, 6673_C>T, 6763_A>G, 6860_A>G, 7392_A>G, 7430_A>G, 7485_A>T

Figure S6. List of panels of unique three-SNP combinations marking >90% of spleen B in m_a.

Panels of unique three-SNP combinations marking >90% of m_a liver T cells

SNP A	SNP B	SNP C
2565_T>A	3699_T>A	4871_A>G, 1006_A>G, 10242_A>G, 11314_T>A, 14995_T>A, 16069_A>T, 2331_A>T, 2415_T>A, 3063_A>T, 3702_A>T, 4575_T>A, 4724_A>T, 6428_T>A, 6656_C>T, 6701_A>T, 6799_A>T, 703_C>G, 7431_A>T, 7455_T>A, 7657_A>G, 7697_T>C, 8329_A>T, 8338_A>T, 8379_T>A,
	11546_T>A	2076_A>G, 4871_A>G, 10242_A>G, 10295_A>G, 11314_T>A, 15251_A>G, 16069_A>T, 2104_T>A, 2492_T>A, 2635_A>G, 3613_T>A, 4575_T>A, 479_A>G, 4856_A>T, 5778_C>T, 6428_T>A, 6656_C>T, 6701_A>T, 7431_A>T, 7455_T>A, 7657_A>G, 7697_T>C, 8194_C>T, 8267_T>A, 8329_A>T, 8387_T>A, 8424_A>T, 8517_T>A, 8577_A>T, 903_A>G

Figure S7. List of panels of unique three-SNP combinations marking >90% of liver T cells in m_a.

Panels of unique three-SNP combinations marking >90% of m_a spleen T cells

SNP A	SNP B	SNP C
11545_G>A	4684_A>G	9276_A>G, 4684_A>T, 4801_A>G, 9207_A>T, 10171_T>C, 11321_A>T, 11453_A>T, 11459_A>G, 13468_T>A, 13899_T>A, 15288_T>A, 4868_A>G, 6658_C>T, 7396_A>T, 7494_A>T, 7550_A>T, 7671_A>G, 8194_C>T, 8246_C>T, 8340_T>A, 8367_T>A, 8471_T>A, 8556_A>G, 9039_A>T
	11423_A>G	9039_A>T, 15289_G>T, 192_A>T, 2076_A>G, 4948_C>T, 5047_A>G, 6557_A>G, 6787_A>G, 7420_A>T, 8439_A>T, 9137_A>T
	3578_T>A	9044_A>G, 11291_A>G, 4700_A>G, 6610_A>G, 9212_A>T, 11160_T>A, 11278_C>T, 2396_A>G, 2470_A>T, 2609_C>T, 3604_T>A, 4863_A>G, 4868_A>G, 6738_A>G, 6797_A>G, 8329_A>T, 8524_A>T, 8598_T>A, 9081_A>G, 9805_A>G
	4378_A>G	8270_C>T, 7497_C>T, 4050_A>T, 5642_A>T, 7411_A>T, 8304_C>T, 8407_A>T, 8424_A>T, 8524_A>T, 9075_A>G, 9166_C>T
4684_A>G	11423_A>G	9072_C>T, 8281_C>T, 9134_C>T, 11453_A>T, 11544_T>G, 2269_T>A, 3702_A>T, 4846_A>G, 4867_A>G, 6557_A>G, 6560_A>G, 6779_A>G, 7420_A>T, 8473_A>T, 8485_C>T, 9039_A>T, 9198_T>A, 9230_A>T, 9296_A>G, 9298_A>G
	3578_T>A	3704_A>G, 4838_A>G, 4949_T>G, 9088_A>G, 9157_C>T, 9167_C>T, 9205_C>G, 11439_A>G, 14962_A>T, 1916_A>T, 2212_A>G, 3285_T>A, 4832_A>G, 4846_A>G, 4950_A>C, 5936_C>T, 7537_C>T, 8305_C>T, 8506_A>G, 9137_A>G, 922_T>C, 9332_A>G
	11294_A>G	8397_A>G, 9131_C>T, 8305_C>T, 9098_A>G, 4685_A>T, 7522_T>A, 7537_C>T, 8388_G>T, 8391_A>G, 9205_C>G, 9226_A>G, 1487_T>A, 14962_A>T, 15288_T>A, 2193_T>A, 3572_A>G, 4701_A>G, 4801_A>G, 4846_A>G, 6779_A>G, 6785_G>A, 7420_A>T, 7476_C>T, 7512_C>T
	4685_A>G	11544_T>C, 4822_A>G, 8341_C>T, 9083_A>G, 9221_A>G, 9296_A>G, 14962_A>T, 15289_G>A, 4377_T>A, 6779_A>G, 7476_C>T, 7482_A>G, 8461_C>T, 9131_C>T, 9226_A>G

Figure S8. List of panels of unique three-SNP combinations marking >90% of spleen T cells in m_a.

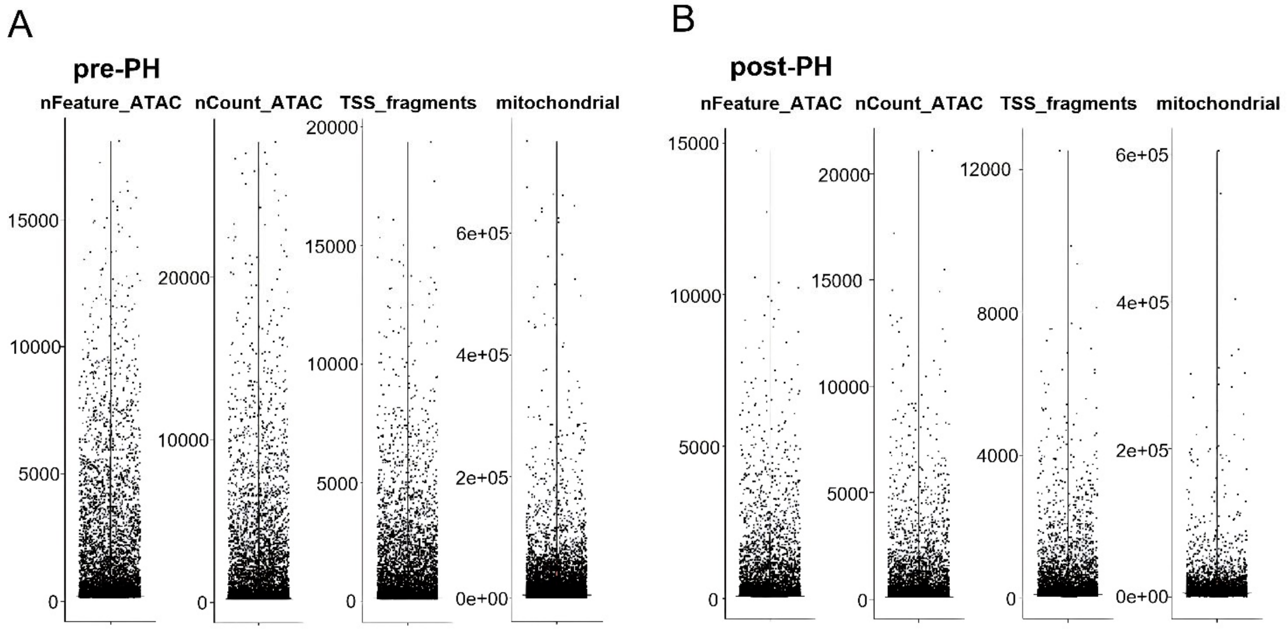

Figure S9. Quality assessment of the mtscATAC data. (A and B) From the left to right, graph showing the unique ATAC-peak number (nFeature_ATAC), captured ATAC peaks reads (nCount_ATAC), ATAC reads around the transcription start site (TSS) (TSS_fragments) and mitochondrial ATAC peak reads (mitochondrial) in the pre-PH (A) and post-PH (B) liver samples from the same mouse.

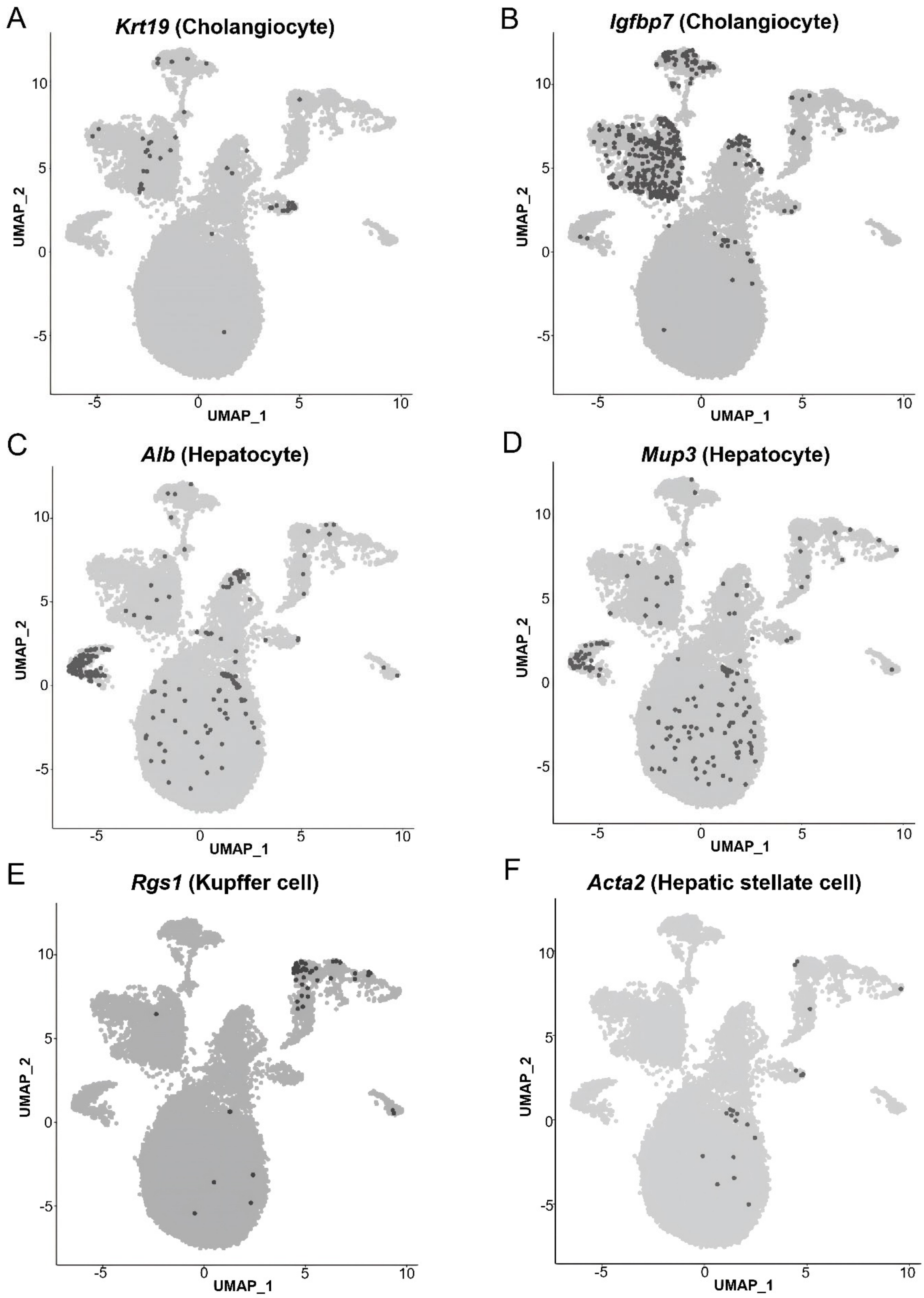

Figure S10. Plotting of feature genes on the mtscATAC UMAP. (A-F) Plot of feature genes (identified based on ATAC peaks) on the pre-PH and post-PH combined UMAP, *Krt19* for cholangiocytes (A), *Igfbp7* for cholangiocytes (B), *Alb* for hepatocytes (C), *Mup3* for hepatocytes (D), *Rgs1* for Kupffer cells (E) and *Acta2* for Hepatic stellate cells (F).

A

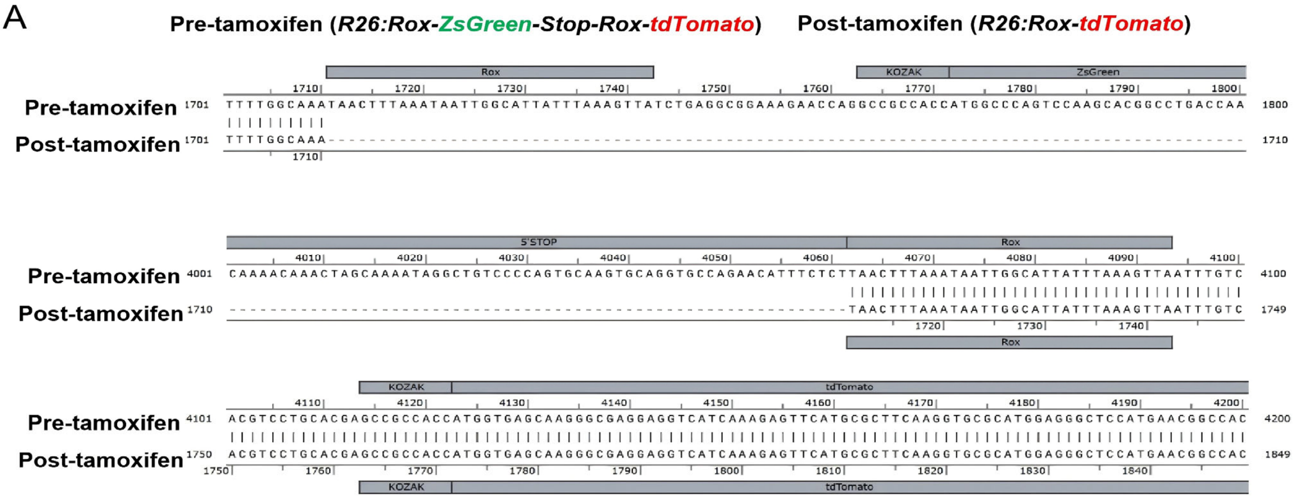

B

SNP A	SNP B	SNP C
13691_T>C	6446_A>C	16095_C>G, 16095_C>A, 16094_C>T, 16094_C>A, 16093_C>A, 16108_T>C, 16112_A>C, 4060_C>A, 6602_C>T, 10182_C>T, 10943_C>T
14935_C>A	4531_A>C	12979_T>G, 13438_C>A, 13773_C>A, 4422_T>C, 7242_C>T,
6634_T>G	16141_T>C	10942_A>G, 10943_C>G, 10944_C>T, 13088_C>A, 15205_C>T, 16094_C>T, 3053_G>A, 4422_T>C, 4800_T>G, 6446_A>C, 6602_C>G, 977_G>A
6689_T>A	7244_C>T	10942_A>G, 12979_T>G, 13773_C>A, 15205_C>T, 16094_C>G, 16105_T>C, 4422_T>C, 9803_G>A
	10939_C>T	10942_A>G, 16094_C>G, 4422_T>C, 6885_T>C, 7242_C>T, 7244_C>T

C

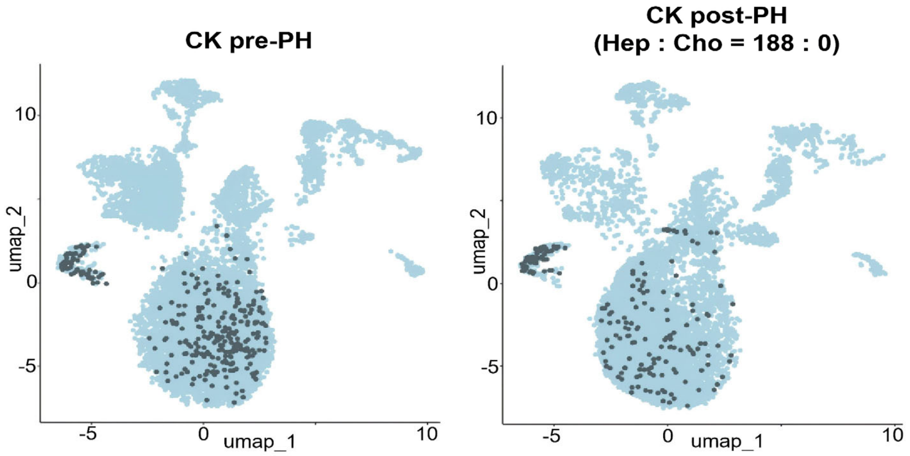

Figure S11. Tracing the fates of pre-PH hepatocytes in the post-PH liver. (A) Sequence before (pre-tamoxifen) and after (post-tamoxifen) excision of *Rox-ZsGreen-Stop-Rox*. (B) List of panels of unique three-SNP combinations collectively marking >90% of hepatocytes in m_a. (C) Feature plot of panels of unique three-SNP combinations marking >90% of the hepatocytes on the UMAP of the pre-PH liver sample of the same mouse used in Figure 9. (D) Plotting of panels of unique three-SNP combinations in (B) onto the UMAP of the post-PH liver sample of the same mouse.
